# Intraoral lipoma with degenerative changes mimicking atypical lipomatous tumor: an immunohistochemical study

**DOI:** 10.4322/acr.2021.413

**Published:** 2022-12-22

**Authors:** Evânio Vilela Silva, Heitor Albergoni Silveira, Beatriz Zamboni Martins Panucci, Kelly Fernanda Molena, Luciana Yamamoto Almeida, Andreia Bufalino, Jorge Esquiche León

**Affiliations:** 1 Universidade Estadual Paulista (Unesp), Araraquara Dental School, Oral Medicine, Department of Diagnosis and Surgery, Araraquara, SP, Brasil; 2 Universidade de São Paulo (USP), Ribeirão Preto Dental School, Oral Pathology, Department of Stomatology, Public Oral Health, and Forensic Dentistry, Ribeirão Preto, SP, Brasil

**Keywords:** Lipoma, Liposarcoma, Mouth, Immunohistochemistry

## Abstract

Lipomas are mesenchymal neoplasms relatively uncommon in the oral cavity. Lipomas can exhibit histopathological features mimicking atypical lipomatous tumors (ALT) or dysplastic lipoma (DL) in the presence of degenerative changes. Relevantly, immunohistochemistry assists in the correct diagnosis. Herein, we present the case of a 54-year-old male with a sessile nodule located on the dorsum of the tongue. The histopathological analysis showed a diffuse, non-circumscribed adipocytic proliferation constituted by cells of variable size containing cytoplasmic vacuoles and displaced nuclei, some resembling lipoblasts supported by fibrous connective tissue stroma. By immunohistochemistry, tumor cells were positive for vimentin, S100, FASN, CD10, and p16. Rb expression was intact. Moreover, CD34, p53, MDM2, and CDK4 were negative. After 2-year of follow-up, no alteration or recurrence was observed. In conclusion, MDM2, CDK4, p53, and Rb immunomarkers can be used reliably to differentiate benign lipoma with degenerative changes from ALT and DL.

## INTRODUCTION

Lipomas are benign mesenchymal neoplasms composed of mature adipocyte cells, relatively uncommon in the oral cavity, representing approximately 4.4% of all benign oral soft tissue tumors. Clinically, lipomas are usually soft, well-circumscribed, mobile, slow growing, and asymptomatic.^1^ In the oral cavity, the buccal mucosa is the most common site, followed by the tongue and lips. Gender preference is variable, affecting patients more frequently between the sixth and seventh decades of life.^2^^,^ 3 Microscopically, lipomas can be classified as classic lipoma, fibrolipoma, angiolipoma, intramuscular lipoma, chondroid lipoma, sialolipoma, and spindle cell lipoma.^4^ However, in sites prone to trauma, such as the oral cavity, lipoma with reactive/degenerative changes may occur. Some of the lipomas with these changes mimic atypical lipomatous tumors (ALT) or dysplastic lipomas (DL), causing difficulty in reaching an accurate diagnosis.^5^^-^ 7 With an apparent predilection for the oral cavity, lipoma with reactive/degenerative changes shows a proliferation of adipocytes of variable size, reactive nuclear atypia, vacuolated histiocytes supported by a fibrous stroma, and fat necrosis.^6^ To date, about 17 cases of intraoral lipoma with degenerative changes have been reported.^6^ All these cases were assessed with MDM2 and CDK4 immunomarkers, with 14 cases being negative and 3 showing weak expression.^6^ Moreover, in a study published 18 years ago, 8 out of 125 lipomas of the oral and maxillofacial region probably correspond to lipoma with degenerative changes.^5^

## CASE REPORT

**Figure 1:**
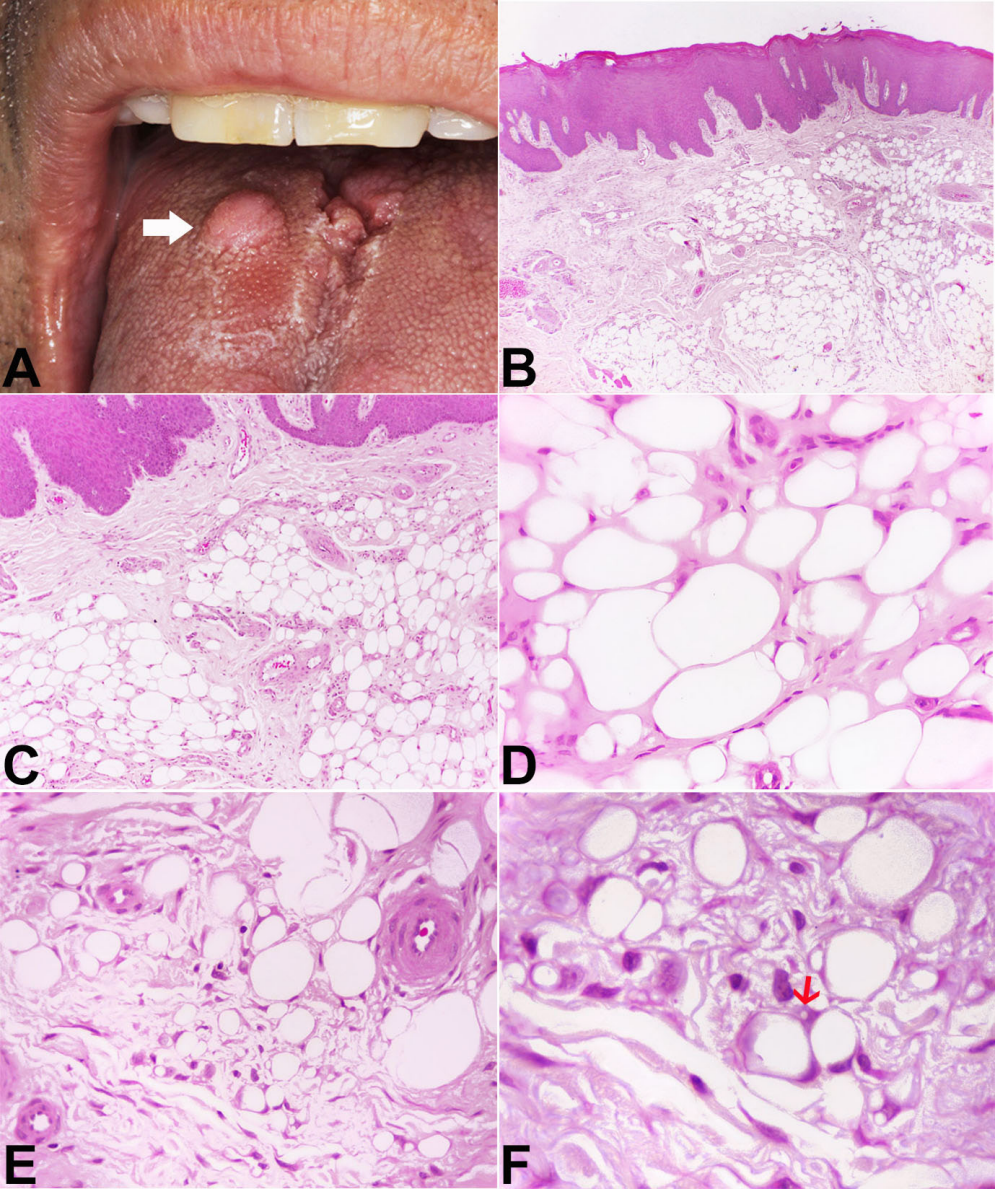
**A -** Clinical presentation of the lesion showing a sessile nodule located on dorsal region of the tongue (arrow); **B to F -** Photomicrographs of the surgical specimen with: **B** and **C** - diffuse, non-circumscribed adipocytic proliferation with variable cell size (H&E, B x4; C x10); **D -** note variably sized adipocytes supported by scarce fibrous stroma, mimicking dysplastic lipoma (H&E; x40); **E -** At the periphery, ring-like cells and vacuolated cells with displaced, slightly hyperchromatic nuclei, some of them with lipoblast-like features, were visualized (H&E, x40); **F -** Close-up view highlighting cellular details. Note uni- or multivacuolated lipoblast-like cells and nuclei with sharply outlined vacuoles, notably Lochkern change (arrow) (H&E, x100).

**Figure 2:**
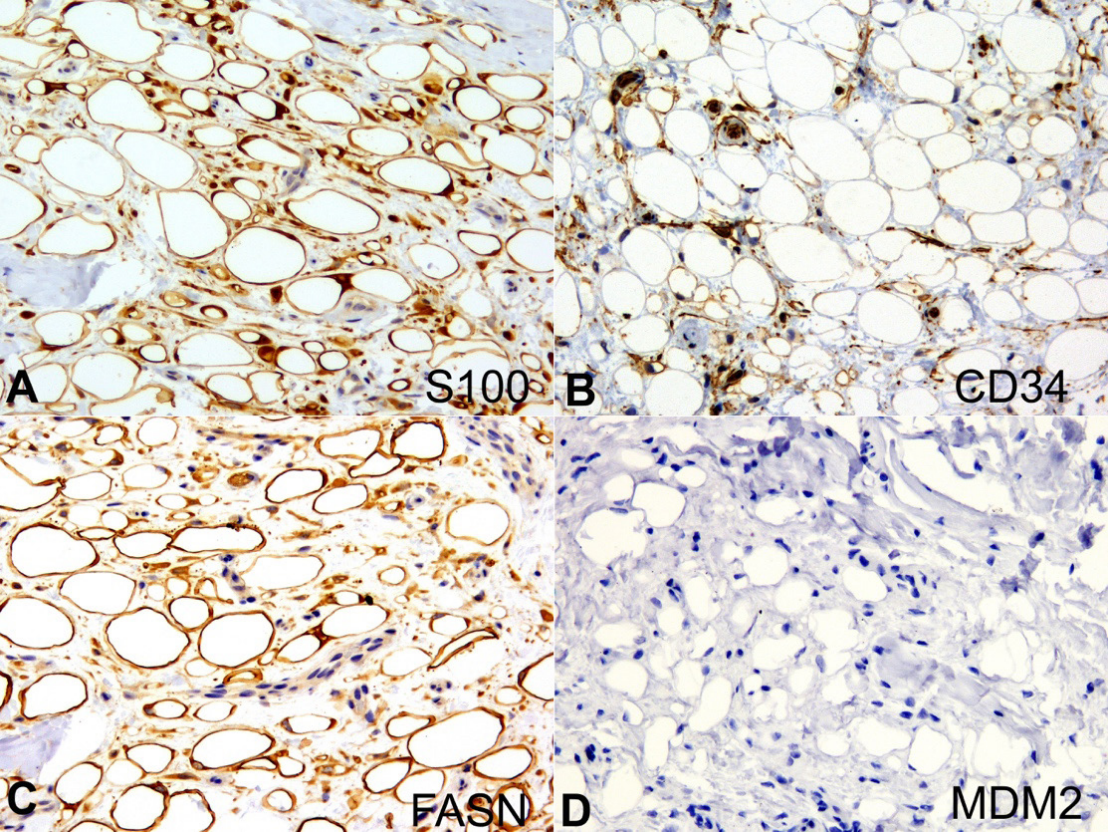
Photomicrographs of the surgical specimen. Immunohistochemical analysis with: **A -** positivity for S100 (x40); **B -** CD34 highlighted the stromal vessels (x40); **C -** FASN evidenced adipocytes varying in size and shape (x40); **D -** negative reaction to MDM2 (x40).

**Figure 3:**
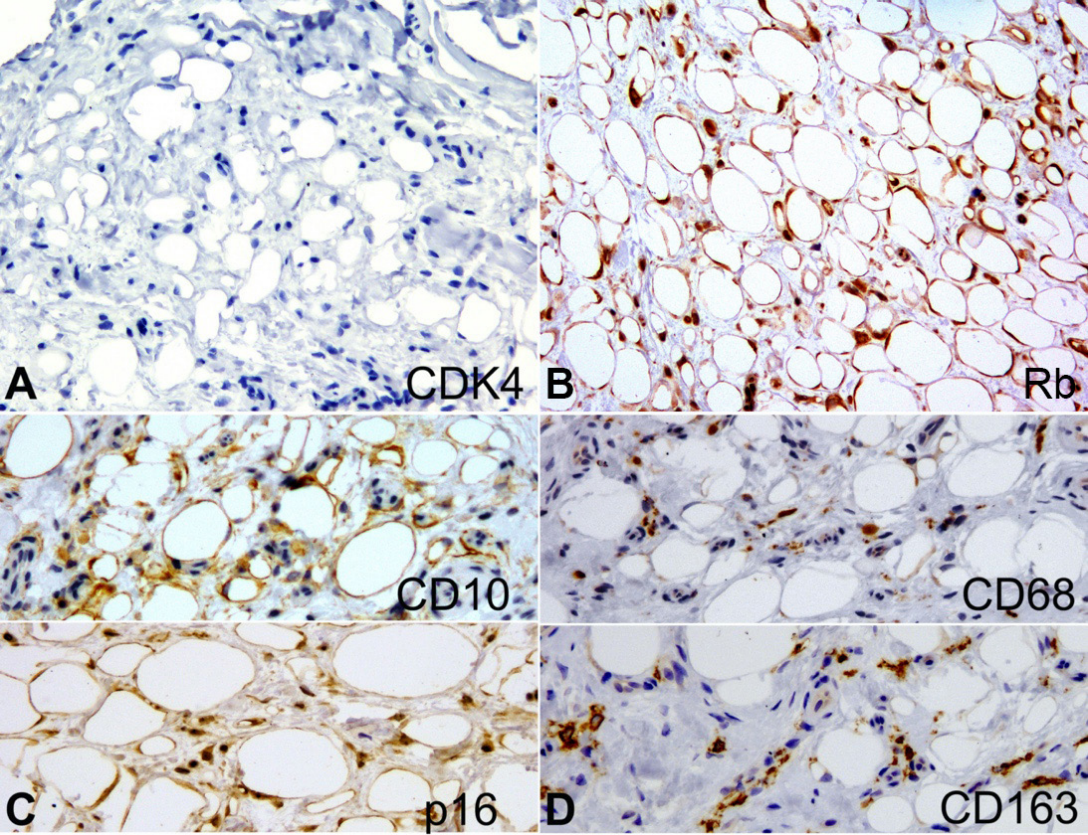
Photomicrographs of the surgical specimen. Immunohistochemical analysis with **A -** Tumor cells were negative to CDK4 (x40), excluding the possibility of ALT; **B -** Rb expression was intact (x40); **C -** CD10 and p16 expression were positive, including the stromal cells (x40); **D -** CD68 and CD163 evidenced numerous stromal histiocytes (x40).

**Figure 4:**
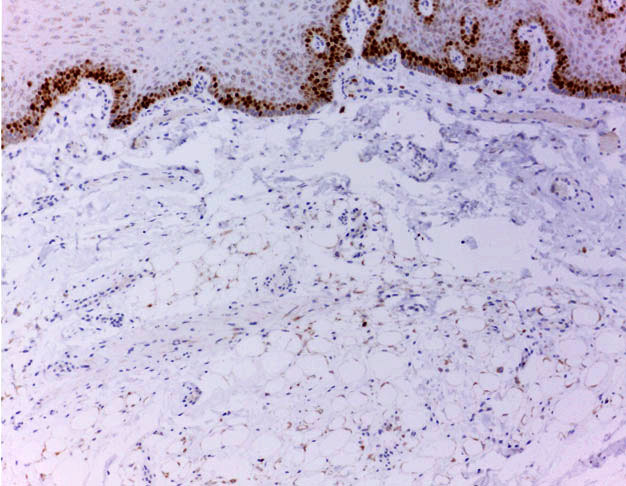
Photomicrograph of the surgical specimen. Rare tumor cells were Ki-67 positive (x10).

A 54-year-old man was referred for evaluation of a nodular lesion on the dorsal surface of the tongue that evolved over several years. The medical history was noncontributory. Intraoral examination revealed a sessile nodule, similar in color to the adjacent normal-appearing oral mucosa, measuring approximately 1.2 × 1.0 × 0.4 cm, located on the dorsal region of the tongue (Figure 1A). An irregular tissue-defined depression or fissures, slightly erythematous on the dorsal tongue’s midline, was also observed and was considered an atypical presentation of median rhomboid glossitis. This lesion improved after cleaning and topical antifungal therapy. The clinical differential diagnosis for the nodular lesion included granular cell tumor, neurofibroma, schwannoma, ectomesenchymal chondromyxoid tumor and lipoma. After total tumor exeresis, the histological examination depicted a diffuse, non-circumscribed adipocytic proliferation by cells of variable size and displaced atypical nuclei in its deepest part, and ring-like cells, Lochkern cells and vacuolated cells, some of them resembling lipoblasts, in the superficial part. These cells were supported by connective tissue stroma containing histiocytes (Figures 1B to 1F). Immunohistochemistry revealed positivity for vimentin, S100, FASN, CD10 and p16, whereas CD34, p53, MDM2 and CDK4 were negative (Figures 2 and 3). Rb expression was intact, and Ki-67 highlighted isolated tumor cells (Figure 4). Moreover, HLA-DR, CD68, CD163, and Factor XIIIA evidenced stromal histiocytes. The histopathological and immunohistochemical findings were consistent with lipoma with reactive/degenerative changes. After 2-year of follow-up, there was no alteration or recurrence.

## DISCUSSION

Benign lipomas are the most common mesenchymal tumors of soft tissue. However, lipomas are relatively uncommon in the oral and maxillofacial regions. In the oral cavity, lipomas may affect various anatomical sites, including the buccal mucosa, tongue, lip, palate, and floor of the mouth.^1^ Some studies indicate that oral lipomas at areas prone to trauma may display degenerative changes^5^^,^ 6 microscopically characterized by variability in adipocyte size, reactive nuclear atypia, fibrosis, increased stromal histiocytes, and fat necrosis, which promote diagnostic pitfalls mimicking ALT or DL.^5^^-^ 7

About 17 cases of intraoral lipoma with degenerative changes have been reported, all of them initially misdiagnosed as ALT.^6^ Most cases revealed a proliferation of adipocytes of varying size, with displaced nucleus resembling atypia, vacuolated histiocytes mimicking lipoblasts, degenerate adipocytes, Lochkern cells, and the presence of mild spindle cell proliferation in a fibrous stroma with focal myxoid degeneration. The affected sites were the tongue, lip, and alveolar mucosa, often prone to trauma. These cases were reevaluated by immunohistochemical reactions to MDM2 and CDK4, nuclear markers, which positivity strongly supports the diagnosis of ALT/well-differentiated liposarcoma (WDL).^6^ The results showed negativity for MDM2 and CDK4 in 14 out of 17 cases, whereas 3 cases showed weak expression of MDM2 and CDK4. By fluorescence in situ hybridization (FISH) analysis for MDM2 amplification, all these 3 cases were negative.^6^ Moreover, histiocytic markers, similar to our case, highlighted vacuolated histiocytes, helping the diagnosis. In the Furlong et al.^5^ study, although an immunohistochemical survey was not carried out, the morphological characteristics in 8 out of 125 cases suggested the possibility of lipoma with degenerative changes.

The ALT/WDL is the most common type of liposarcoma (40-45% of all liposarcomas), usually affecting the retroperitoneum and thighs of middle-aged adults, being rare in the oral cavity.^8^ It is important to mention that the terminology “ALT” and “WDL” is based on a tumor's location and primarily relate to resectability. For tumors located in the periphery, such as the oral cavity, complete resection is generally curative, without risk of metastasis, for which “ALT” is preferred. On the other hand, “WDL” is more appropriate for deep-seated tumors, such as the retroperitoneum and mediastinum, where the positive margins, local recurrence and dedifferentiation are significantly increased.^6^^,^ 8

To date, 73 cases of intraoral ALT/WDL have been reported, slightly predominant in women with a mean age of 55. The most common oral location was the tongue (38 cases), followed by the lip, floor of the mouth, gingiva, and palate. Most of these cases appeared as a circumscribed, indolent, asymptomatic, firm, or elastic submucosal mass. The mean size of the tumor was 2.5 cm, with a mean duration of 30 months. Recurrence was reported in 9 cases, with no metastases or deaths.^5^^,^ 8 ^,^ 9

Histologically ALT/WDL are characterized by presenting mature fat with variably sized adipocytes and spindle cells with enlarged, hyperchromatic nuclei located between adipocytes and/or within dense fibrous septa and in a perivascular distribution. However, these classic features are not always present, and, as such, without the help of immunohistochemistry, ALT/WDL may not be properly diagnosed.^8^^,^ 10 ^-^ 12 The differential diagnosis of ALT/WDL includes intramuscular lipoma, spindle cell lipoma, pleomorphic lipoma, chondroid lipoma, lipoma with fat necrosis and/or Lochkern cells, and atrophy of fat.^8^^,^ 9 Relevantly, only 6 out of 73 intraoral cases of ALT/WDL reported additional analysis of MDM2 and/or CDK4,^10^^-^ 12 which should be considered for diagnosis. Thus, it should not be surprising if some of these cases represent benign lipoma with reactive/degenerative changes. For the correct diagnosis of ALT/WDL, the MDM2 and CDK4 immunomarkers, and MDM2 amplification assessed by FISH, have been used to increase the diagnostic accuracy.^6^^,^ 9 Notably, the nuclear overexpression of MDM2 and CDK4 in ALT/WDL results from the 12q13-15 chromosomal amplification in ring or giant marker chromosomes. Benign lipoma may have various cytogenetic findings, including 12q13-15 rearrangement targeting HMGA2, 13q deletion, or 6p21-23 rearrangement but does not overexpress MDM2/CDK4.^6^ An interesting study focusing on immunoexpression of MDM2/CDK4 in ATL/WDL and benign lipomatous lesions^13^ showed that MDM2 and CDK4 were detected in 25/56 and 23/56 ALT/WDL, respectively, corresponding to a sensitivity of 45% and 41% and a specificity of 98% and 92%. In contrast, in benign lipomatous lesions MDM2 and CDK4 expression occurred in 2/125 and 10/117 cases, respectively.^13^ In the current case, a positive expression of vimentin, S100, CD10, and FASN highlighted their mesenchymal and fatty nature. While p16 expression was initially proposed as a successful diagnostic marker with selective expression in malignant adipocytic tumors, it has been recognized that p16 can be detected in both benign and malignant adipocytic tumors, as observed in the current case.^14^

The DL, a recently described fatty tumor, has a distinctive morphology independent of the anatomic site. This tumor shows male predilection, mainly affecting the neck and back. No case involving the oral cavity has been reported to date.^7^^,^ 15 Morphologically, DL tends to form well-demarcated subcutaneous masses with striking adipocytic size variation and patchy single-cell fat necrosis. Spindle stromal cells and collagenous matrix tend to be scant or absent. Adipocytes with one or more atypical nuclei are always present, but they may be sparse and difficult to depict at low magnification. On close inspection, the atypical adipocytes have nuclei variably enlarged with coarsened chromatin, small nucleoli, and frequently, focal Lochkern change.^6^ In these cases, p53 overexpression, loss of Rb expression, including RB1 gene abnormalities, low MDM2 reactivity, and lack of MDM2 gene amplification by FISH, are observed in most tumor cells.^7^^,^ 15 The current case presented DL-like microscopical features in the deepest part of the lesion; however, focal areas presented mild adipocytic size variation. Moreover, p53 was negative, and the expression of Rb was intact.

It should be noted that benign symmetrical lipomatosis (BSL), a rare disorder characterized by diffuse, multiple symmetric, and non-encapsulated fat tissue masses, often affecting the face, neck, and upper trunk can also manifest intraoral involvement. Approximately 8 cases of BSL with lingual involvement have been reported.^16^ Unlike lipoma with reactive/degenerative changes, these cases present a homogeneous proliferation in shape and size of adipocytes, with an indistinct margin between the adipose tissue and skeletal muscle, which makes the surgical resection troublesome with a relatively high rate of recurrence or regrowth.^16^

The treatment of choice for benign lipoma is conservative surgery, and recurrences are infrequent.^1^ Similarly, for DL, simple local excision is recommended; however, recurrences can occur in about 4% of the cases.^7^ For ALT, the optimal treatment is wide surgical excision with free margins, with the risk of recurrence or metastasis being very low or absent; notwithstanding, continuous and rigorous monitoring is necessary.^8^^,^ 10 ^,^ 12 In this context, it is important to distinguish lipoma with reactive/degenerative changes from ALT and DL.

## References

[1] Studart-Soares EC, Costa FWG, Sousa FB, Alves APNN, Osterne RLV (2010). Oral lipomas in a Brazilian population: a 10-year study and analysis of 450 cases reported in the literature. Med Oral Patol Oral Cir Bucal.

[2] Manor E, Sion-Vardy N, Joshua BZ, Bodner L (2011). Oral lipoma: analysis of 58 new cases and review of the literature. Ann Diagn Pathol.

[3] Santos JL, Ocamoto EA, Almeida LY, Teixeira LR, Ribeiro-Silva A, León JE (2017). Low-fat plexiform spindle cell lipoma with prominent myxoid stroma: an unusual oral presentation and immunohistochemical analysis. J Craniofac Surg.

[4] Rocha AFL, Miotto LN, Ferrisse TM (2019). Low-fat and fat-free spindle cell lipomas in the oral cavity: immunohistochemical analysis and review of the literature. J Cutan Pathol.

[5] Furlong MA, Fanburg-Smith JC, Childers EL (2004). Lipoma of the oral and maxillofacial region: site and subclassification of 125 cases. Oral Surg Oral Med Oral Pathol Oral Radiol Endod.

[6] Stojanov IJ, Mariño-Enriquez A, Bahri N, Jo VY, Woo SB (2019). Lipomas of the oral cavity: utility of MDM2 and CDK4 in avoiding overdiagnosis as atypical lipomatous tumor. Head Neck Pathol.

[7] Michal M, Agaimy A, Contreras AL (2018). Dysplastic Lipoma: A distinctive atypical lipomatous neoplasm with anisocytosis, focal nuclear atypia, p53 overexpression, and a lack of MDM2 gene amplification by FISH; a report of 66 Cases demonstrating occasional multifocality and a rare association with retinoblastoma. Am J Surg Pathol.

[8] Piperi E, Tosios KI, Nikitakis NG (2012). Well-differentiated liposarcoma/atypical lipomatous tumor of the oral cavity: report of three cases and review of the literature. Head Neck Pathol.

[9] Nili F, Baghai F, Aghai A, Etebarian A (2016). Well-differentiated liposarcoma of the floor of the mouth: report of a rare case and review of the literature. J Oral Maxillofac Pathol.

[10] Ohta K, Yoshimura H, Matsuda S, Imamura Y, Sano K (2020). Oral liposarcoma in elderly: case report and literature analysis. Medicine.

[11] Nikitakis NG, Lopes MA, Pazoki AE, Ord RA, Sauk JJ (2001). MDM2+/CDK4+/p53+ oral liposarcoma: case report and review of the literature. Oral Surg Oral Med Oral Pathol Oral Radiol Endod.

[12] Laco J, Mentzel T, Hornychova H, Kohout A, Jirousek Z, Ryska A (2009). Atypical lipomatous tumors of the tongue: report of six cases. Virchows Arch.

[13] Clay MR, Martinez AP, Weiss SW, Edgar MA (2016). MDM2 and CDK4 immunohistochemistry: should it be used in problematic differentiated lipomatous tumors? A new perspective. Am J Surg Pathol.

[14] Kammerer-Jacquet SF, Thierry S, Cabillic F (2017). Differential diagnosis of atypical lipomatous tumor/well-differentiated liposarcoma and dedifferentiated liposarcoma: utility of p16 in combination with MDM2 and CDK4 immunohistochemistry. Hum Pathol.

[15] Evans HL (2016). Anisometric cell lipoma: a predominantly subcutaneous fatty tumor with notable variation in fat cell size but not more than slight nuclear enlargement and atypia. AJSP Rev Rep..

[16] Mayo Yáñez M, González Poggioli N, Álvarez-Buylla Blanco M, Herranz González-Botas J (2018). Benign symmetric lipomatosis with lingual involvement: case report and literature review. J Stomatol Oral Maxillofac Surg.

